# Transcriptome profiling, physiological, and biochemical analyses provide new insights towards drought stress response in sugar maple (*Acer saccharum* Marshall) saplings

**DOI:** 10.3389/fpls.2023.1150204

**Published:** 2023-04-19

**Authors:** Lungowe Mulozi, Amaranatha R. Vennapusa, Sathya Elavarthi, Oluwatomi E. Jacobs, Krishnanand P. Kulkarni, Purushothaman Natarajan, Umesh K. Reddy, Kalpalatha Melmaiee

**Affiliations:** ^1^Department of Agriculture and Natural Resources, Delaware State University, Dover, DE, United States; ^2^Department of Biology and Gus R. Douglass Institute, West Virginia State University, Institute, WV, United States

**Keywords:** *Acer saccharum*, sugar maple, transcriptome, drought, stress, physiology, gene expression

## Abstract

Sugar maple (*Acer saccharum* Marshall) is a temperate tree species in the northeastern parts of the United States and is economically important for its hardwood and syrup production. Sugar maple trees are highly vulnerable to changing climatic conditions, especially drought, so understanding the physiological, biochemical, and molecular responses is critical. The sugar maple saplings were subjected to drought stress for 7, 14, and 21 days and physiological data collected at 7, 14, and 21 days after stress (DAS) showed significantly reduced chlorophyll and Normalized Difference Vegetation Index with increasing drought stress time. The drought stress-induced biochemical changes revealed a higher accumulation of malondialdehyde, proline, and peroxidase activity in response to drought stress. Transcriptome analysis identified a total of 14,099 differentially expressed genes (DEGs); 328 were common among all stress periods. Among the DEGs, transcription factors (including NAC, HSF, ZFPs, GRFs, and ERF), chloroplast-related and stress-responsive genes such as peroxidases, membrane transporters, kinases, and protein detoxifiers were predominant. GO enrichment and KEGG pathway analysis revealed significantly enriched processes related to protein phosphorylation, transmembrane transport, nucleic acids, and metabolic, secondary metabolite biosynthesis pathways, circadian rhythm-plant, and carotenoid biosynthesis in response to drought stress. Time-series transcriptomic analysis revealed changes in gene regulation patterns in eight different clusters, and pathway analysis by individual clusters revealed a hub of stress-responsive pathways. In addition, qRT-PCR validation of selected DEGs revealed that the expression patterns were consistent with transcriptome analysis. The results from this study provide insights into the dynamics of physiological, biochemical, and gene responses to progressive drought stress and reveal the important stress-adaptive mechanisms of sugar maple saplings in response to drought stress.

## Introduction

Sugar maple (*Acer saccharum* Marshall) is a keystone tree species abundantly distributed in the northeastern forests of the United States and eastern Canada (Horsley et al., 2002). It is broadly known for its vibrant fall colors, syrup, and hardwood, and plays a vital role in its native ecosystems (Godman et al., 1990; Lucash et al., 2012). The maple industry contributed $147 million to the US economy in 2016 (Snyder et al., 2019). The United States was the second major producer of maple syrup worldwide, with a total production of ~4.24 million gallons in 2019. The state of Vermont produced more than 1.5 million gallons in 2021 and was the top maple syrup producer state in the United States. New York was the second leading producer, with a production of about 647 thousand gallons in 2021 (Shahbandeh, 2021). The global market value for maple syrup was at an all-time high of $1.24 billion in 2018 and is anticipated to increase by 2023 with a compound annual growth rate of 7% to $1.7 billion. Even though the United States is the second highest producer of maple syrup, it imports more than it produces each year. The import rate has increased over the past 40 years, from 607,000 in 1975 to 6.1 million in 2018. In 2018, the United States represented 50% ($182 million) of the world’s import value, with 100% of the imports coming from Canada^1^. The current demand for maple products worldwide and in the United States is increasing steadily (Farrel, 2021).

Drought being the inevitable factor, significantly affects the plant morphological, physiological, biochemical, and molecular features with an adverse influence on growth, photosynthetic capacity, membrane fluidity, and antioxidant machinery. At cellular and molecular levels, a significant effect of drought stress is seen on turgor pressure maintenance, membrane stability, sugar metabolism, respiration, interruption of metabolic and biochemical pathways, protein degradation, enzyme dysfunctions, induction of stress signaling, and sensitive genes (Farooq et al., 2009; McDowell, 2011). Plants cope with drought stress through various mechanisms, such as morphological by water mining with profound root systems, conservation by waxes, reduced leaf size, stay green nature, growth and development, and physiological mechanisms by maintaining the photosynthetic machinery, assimilate partitioning, stomatal regulation, turgor maintenance (Anjum et al., 2011; Sajeevan et al., 2017; Seleiman et al., 2021; Venkatesh et al., 2022). Biochemical mechanisms involved osmolyte and secondary metabolite production, enzymatic functions, maintenance of metabolic, protein, and lipid biosynthetic pathways, and molecular mechanisms involved in the maintenance of RNA and DNA synthesis and stress signaling, cascade, and regulatory genes expression, especially the stress-induced transcription factors, and stress-responsive downstream genes (Mickelbart et al., 2015; Kiranmai et al., 2018; Lokesh et al., 2019; Zhang et al., 2022).

Due to global climate change, the frequency and intensity of drought have continuously increased (Dai, 2013; Lima et al., 2015; Gugger et al., 2017). In plants, the ability to respond to increasing drought conditions will be particularly important if the current climate scenario continues (Gugger et al., 2017). Episodes of sugar maple decline have arisen sporadically since the early 1900s due to climate change. The major factors affecting decline include defoliation by insects, intense drought, elevated seasonal temperatures, winter freezing injury, and early autumn frosts (Horsley et al., 2000; Wong et al., 2009; Rogers et al., 2017; Oswald et al., 2018; Rogers, 2020). Severe and recurrent droughts are a significant threat to the productivity of sugar maple and account for unusual mortality in several states in the United States (Horsley et al., 2002; Oswald et al., 2018). Drought stress-induced changes were reported in crown growth, soluble sugars, net photosynthesis, leaf color, nutrient limitation during the autumn, and anthocyanin production in sugar maple plants (Schaberg et al., 2003; Wong et al., 2009). The drought stress in sugar maple trees reduces the chlorophyll content, and growth and induces early abscission of leaves (Carlson, 2009).

Although observational studies and reports have indicated that drought is one of the major factors responsible for sugar maple decline, no comprehensive physiological and genetic studies have been conducted to substantiate this theory (Harmon et al., 2017). Understanding the mechanisms involved in plants coping with drought stress has been a central topic in plant physiology for decades (Bray, 1997: Gugger et al., 2017; Hauer et al., 2021). Despite knowing the significant effect of drought stress on sugar maple survival, plant growth, and development, limited information is available on plant physiological processes, and the molecular mechanisms in response to drought stress remain unexplored. Hence, investigating the molecular mechanisms in response to drought stress in sugar maple offers a possibility for understanding stress adaptation mechanisms.

Next-generation sequencing has become a powerful platform to elucidate molecular mechanisms in many research fields due to its rapidity and cost-effectiveness (Gao et al., 2018; Callwood et al., 2021; McEvoy et al., 2022). The comprehensive data generated facilitates the development of genetic analyses and functional genomics studies between plant species, specifically for non-model plants and tree species (Kulkarni et al., 2021). Transcriptome studies are a powerful tool to provide valuable molecular and genetic information, including the discovery of molecular markers and novel candidate genes; insight into genetic networks, transcriptional and post-transcriptional regulation; and pathways involved in drought stress tolerance in many plant species (Aranda et al., 2012; Zhang et al., 2020; Song et al., 2022). Additionally, a meta-analysis of large transcriptomic datasets can reveal the key genetic resources responsible for complex tolerant traits, which are useful in crop improvement programs (Baldoni, 2022). In particular, global transcriptomic analysis has been widely used to understand drought stress response in various tree species, such as oak (Spieß et al., 2012; Gugger et al., 2017), pine (Fox et al., 2018), and beech (Müller et al., 2017).

The present study investigated the physiological, biochemical, and transcriptome changes of sugar maple to drought stress. Potential responsive genes were identified using RNAseq. This study describes the first genome-wide expression profiles of sugar maple in response to drought stress. The results from this study provide a comprehensive understanding of the molecular, physiological, and biochemical mechanisms of sugar maple plants in response to drought stress and help identify potential candidate genes for drought resistance, which can be used in sugar maple breeding programs for improving drought stress tolerance.

## Materials and methods

### Drought-stress experiments and sample collection

Sugar maple saplings were obtained from the Cold Stream Farms Nursery (Free Soil, MI, USA). The saplings were transplanted into medium-sized pots (15 cm deep) filled with peat moss and perlite. They were maintained under ambient environmental conditions, regularly watered, and fertilized for 12 months to establish and obtain sufficient biomass before undergoing drought stress. Then 28 uniform-sized saplings were transferred into the greenhouse and kept for one month for acclimatization. The conditions in the greenhouse were an average temperature of 28 ± 2°C with an average photoperiod of 14 h/day provided by natural sunlight. The plants were randomly divided into four groups: one control and three different drought stress treatments. Each group contained seven plants that were tagged with four different colored tags to indicate the four different treatments. Drought stress was imposed in May 2020. The control treatment plants were regularly irrigated every day with 500 ml water. For drought stress, a group of plants was left unwatered for 21 days (21 days after stress imposition [21DAS]), another group for 14 days (14DAS), and another group for 7 days (7DAS). The stress was imposed ahead of the corresponding number of days of treatment so that the experiments could be ended on the same day. Physiological measurements were taken, and leaf samples were collected at the same time at the end of the experiments. This sampling was based on the fact that all plants will undergo the same environmental and growth changes except for the water stress they receive. There were seven plants for each treatment, among them, three plants were utilized for biochemical analysis and four plant for RNAseq analysis for each treatment as biological replicates. The topmost fully expanded young fresh leaf samples were collected from the selected plants and were frozen in liquid nitrogen and stored at -80°C.

### Soil volumetric water content measurements

The volumetric water content of the soil (moisture content in peat moss) was measured by using a hand-held FieldScout Time Domain Reflectometer (TDR) 100 Soil Moisture Meter (Spectrum Technologies, Aurora, IL, USA). Moisture content readings were taken in three different spots in the pot, and the final readings were the average of the three readings. The readings were taken from both control and drought stress pots (7, 14, and 21DAS) (Gebre & Earl, 2021).

### Physiological and biochemical measurements

Physiological parameters were measured on three randomly selected leaves from each plant (one on the top, one in the center, and one at the lower base). For each leaf, measurements were taken three times on different spots of the leaf to obtain an average reading. These leaves were labeled, and the Normalized Difference Vegetation Index **(**NDVI) was measured similarly by using a handheld PolyPen RP 410 device (Qubit systems, Canada) (Wu et al., 2019). The chlorophyll index was measured by using the Soil and Plant Analysis Development (SPAD) metric (Konica Minolta, Japan) (Vennapusa et al., 2021).

Proline content was estimated according to the modified method (Vemanna et al., 2017; Siddique et al., 2018; Bissiwu et al., 2022) from Bates et al. (1973). The frozen leaf samples were ground by adding 500 μL of 3% sulfosalicylic acid in microcentrifuge tubes using a Fisher scientific bead mill 24. The samples were centrifuged for 5 min at 14,000 rpm by using a centrifuge (5417R, Eppendorf, Hamburg, Germany). Then, 100 μL supernatant was collected and added to the reaction mixture containing 100 μL of 3% sulfosalicylic acid, 200 μL glacial acetic acid, and 200 μL acidic ninhydrin and incubated at 96°C for 60 min. The reaction was terminated by keeping the samples on ice for 5 min; after cooling with 600 μL toluene added to the incubated samples and brief vortexing for 5 min for separating the samples, the upper aqueous phase of the 200-μL sample was used for spectrophotometer analysis. The absorbance was measured at 520 nm by using a 96-well microplate reader. The proline content in samples was determined with a standard curve using L-proline and calculated on a fresh weight basis and expressed as μg/g fresh weight (FW).

Malondialdehyde (MDA) content was estimated by using Lipid Peroxidation (MDA) Assay Kit (Munich, Germany). A homogenate of leaf samples (50 mg) was prepared in 500 μL MDA lysis buffer containing 3 μL butylated hydroxytoluene (BHT 100x). Samples were centrifuged at 13,000 rpm for 10 min to separate solid particles from the supernatant. A clear supernatant of 200 μL was added to 600 μL of the Thiobarbituric acid (TBA, 20%) solution and incubated at 95°C for 60 min to form the MDA-TBA adducts. Then the samples were cooled to room temperature in an ice bath for 10 min. Finally, 200 μL of the reaction mixture from plant samples, blank, and standards was transferred to 96-well plates for analysis, and absorbance of the supernatant was measured at 532 nm. Both standard and sample values were corrected by subtracting the blank value from all readings. The MDA concentration in treatment samples was estimated in µmoles/g FW with a standard curve plot according to guidelines given in the protocol.

### Total RNA isolation, library preparation, poly-A selection, and HiSeq sequencing

Total RNA was isolated from leaf tissues of four biological replicates for each treatment by using Qiagen RNeasy plus a universal mini kit following the manufacturer’s instructions (Qiagen, Hilden, Germany). Total RNA was quantified using NanoDrop 2000C (Thermo Scientific, Waltham, MA, USA), and quality was checked using the Qubit 2.0 Fluorometer (Invitrogen, Carlsbad, CA, USA). The integrity (RIN) was studied using Bioanalyzer, Agilent TapeStation 4200 (Agilent Technologies, Palo Alto, CA, USA). RNAseq libraries were prepared using the NEBNext Ultra RNA Library Prep Kit for Illumina with the manufacturer’s guidelines (NEB, Ipswich, MA, USA). Briefly, mRNAs were initially enriched with Oligod (T) beads. Enriched mRNAs were fragmented for 15 min at 94°C. First- and second-strand cDNA was synthesized. cDNA fragments were end-repaired and adenylated at 3’ends, and universal adapters were ligated to cDNA fragments, followed by index addition and library enrichment by PCR with limited cycles. The sequencing library was validated on the Agilent TapeStation (Agilent Technologies, Palo Alto, CA, USA) and quantified by using the Qubit 2.0 Fluorometer (Invitrogen, Carlsbad, CA, USA) as well as by quantitative PCR (KAPA Biosystems, Wilmington, MA, USA). The sequencing libraries were clustered on three lanes of a flow cell, and the flow cell was loaded on the Illumina HiSeq instrument (4000) according to the manufacturer’s guidelines (Illumina. San Diego, CA, USA). The samples were sequenced by using a 2x150-bp paired-end configuration. Image analysis and base calling involved using HiSeq Control Software (HCS). Raw sequence data (.bcl files) generated from Illumina HiSeq were converted into fastq files using Illumina’s bcl2fastq 2.17 software (Illumina, USA).

### *De novo* transcriptome assembly, read mapping, and sequence annotation

The adapters and low-quality sequencing reads (Q <30) were trimmed using Trimmomatic v.0.39 (Bolger and Giorgi, 2014; Natarajan et al., 2021). The quality-filtered reads were mapped to the reference *de novo* transcriptome generated with Trinity assembler and clustering with CD-HIT-EST using default parameters. The completeness and integrity of the *de novo* transcriptome assembly were assessed using Benchmarking Universal Single-Copy Orthologs (BUSCO) analysis and embryophyta_odb10 dataset (Simão et al., 2015). The read count table was created by using RSEM and Bowtie2. The differentially expressed genes (DEGs) among pair-wise stress treatment combinations were identified using the R package DESEq2 (Love et al., 2014). The minimum log 2_-_fold change = 1 and false discovery rate (FDR) cutoff 0.05 were used to filter the DEGs. Gene Annotation and Gene Ontology (GO) enrichment analyses were performed using the Omicsbox tool (https://www.biobam.com/omicsbox/). The unigenes were annotated against databases of NR, InterPro (UniProtKB/Swiss-Prot), COG, KOG, and CDD) Pathway mapping involved using the Kyoto Encyclopedia of Genes and Genomes (KEGG) database (Natarajan et al., 2021) and the Pathview web tool (Luo et al., 2017). Time-series gene expression and co-expressed gene clusters with similar expression patterns across conditions were analyzed using maSigPro (Wan et al., 2017).

### Quantitative real-time PCR

Quantitative real-time PCR (RT-qPCR) analysis was performed to validate the expression of DEGs identified in the RNAseq analysis. Total RNA for sugar maple leaves from all drought stress treatments and control samples was extracted using the Spectrum Plant Total RNA Kit (Sigma-Aldrich, St Louis, MO, USA). The RevertAid First Strand cDNA Synthesis Kit was used for cDNA synthesis (Thermo Scientific, Waltham, MA, USA). Primers for the selected genes were designed by Primer 3 software and custom-synthesized from IDT (Integrated DNA Technologies, Coralville, IA, USA). A list of the primers used for the validation is in **Table S1**. Quantitative gene expression analysis used the RT-qPCR system (Step Plus one, Applied Biosystems, Waltham, MA, USA). Four biological and three technical replicates were used for each treatment. The RT-qPCR reaction mixture contained 8 μL of Power track SYBR Green master mix (Applied Biosystems, Waltham, MA, USA), 2 μL of each of the forward and reverse primer (5 μM), and 20 ng template cDNA (2 μL) for a final volume of 14 μL. The RT-qPCR program was set at 95°C for 5 min, followed by 35 cycles of 95°C for 30 s and 60°C for 30 s. Relative gene expression was quantified by the 2^-ΔΔCt^ method, and fold change in gene expression under stress conditions was calculated relative to control samples (Livak and Schmittgen, 2001; Vennapusa et al., 2020). The gene expression was normalized to the expression of *B-tubulin* and *actin* as the reference genes.

### Estimation of peroxidase activity

The peroxidase activity (POD) was measured using the guaiacol peroxidase protocol (Cavalcanti et al., 2004). The frozen leaf material (1.0 g) was homogenized in 3 ml of ice-cold 100 mM potassium phosphate buffer with pH 6.8 containing 0.1 mM EDTA using mortar and pestle. The homogenate samples were allowed for 5 min and filtered through cheesecloth to remove the plant debris and centrifuged at 16 000 g for 15 min at 4˚C, and all the steps were carried out on the ice. The supernatant was used as a crude enzyme extract for the assay. The activity of the guaiacol peroxidase (POD) enzyme was determined by adding 25 µl of the crude enzyme extract to 2 ml of an assay buffer containing 50 mM K-phosphate buffer with pH 6.8, 20 mM guaiacol, and 20 mM H_2_0_2_. The assay samples were incubated at 30˚C for 10 min, and the reaction was stopped by adding 0.5 ml of 5% (v/v) H2SO4, and the absorbance was measured at 480 nm. One unit of POD was expressed as the change of 1.0 absorbance unit per ml enzymatic extract and defined as units of enzyme activity per g fresh weight per min (UA g^-1^ FW min^-1^).

### Statistical analysis

One-way ANOVA was used to estimate the significant differences between drought stress treatments on various physiological and biochemical traits and gene expression studies (Pandian et al., 2020). The R package ggplot2 was used to generate bar graphs (Wickham and Wickham, 2007; Vennapusa et al., 2022). Fisher’s LSD test was used to separate the mean of significant treatments at P ≤ 0.05 by using the R package agricolae (de Mendiburu, 2020; Pandian et al., 2021).

## Results

We investigated the physiological, biochemical, and molecular responses of sugar maple to drought stress. Transcriptome analysis was performed, and a *de novo* reference genome was assembled to identify DEGs between control and drought-stressed samples.

### Morpho-physiological responses of sugar maple to drought stress

The drought stress effect on sugar maple was assessed by imposing drought stress for 7, 14, and 21 days. The control plant pots (watered every day) maintained a soil volumetric water content of 45%; 7-day stressed plant pots maintained a soil moisture content of about 29%, and 14- and 21-day stressed plant pots maintained a soil moisture content of about 17% (**Figure S1**). The control plants grew normally and maintained the stay-green nature, with the regeneration of young leaves (**Figure S2**). The 7-day drought-stressed plants showed relatively fewer yellow symptoms on leaves as compared with 14- and 21-day stressed plants. The plants exposed to 14 days of stress showed distinct yellowish symptoms and some necrotic spots as compared with 7-day stressed plants. The plants exposed to 21 days of stress showed severe yellow and necrotic symptoms with wilting and shoot meristem burning at the tips as compared with all other stress treatments (**Figure S2**).

**Figure 1:**
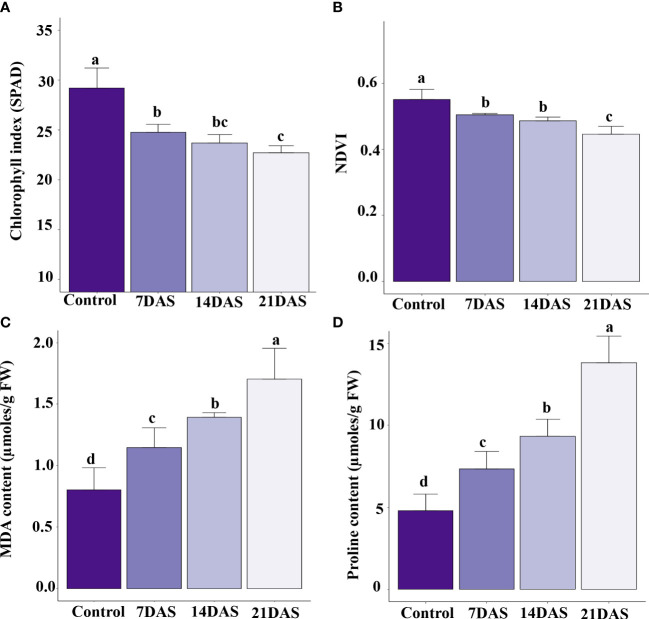
Physiological and biochemical changes in sugar maple saplings in response to drought stress: Sugar maple plants were subjected to drought stress for 7, 14, and 21 days and data were analyzed by comparison with the control samples. **(A)** Chlorophyll index (SPAD), **(B)** NDVI, **(C)** MDA, and **(D)** proline content. Different alphabetic letters on top of the error bar graphs indicate statistically significant differences between the stress periods (p ≤ 0.05). SPAD: Soil and Plant Analysis Development; NDVI: Normalized Difference Vegetation Index; MDA: malondialdehyde.

The drought stress effect on chlorophyll content and photosynthesis in sugar maple was assessed with SPAD and NDVI measurements. All leaf-level physiological variables showed significant variation (p ≤ 0.05) with different times of drought stress exposure. The chlorophyll content represented by SPAD units showed a significant effect of drought stress. The control plants maintained a higher chlorophyll index, and a higher reduction was observed at 21DAS (22.7) compared to other stress treatments (7DAS [24.7] and 14DAS [23.7]; **Figure 1A**). The NDVI in the drought-subjected leaf samples was significantly affected by drought stress. The control plants maintained higher NDVI (0.55), and a decreasing trend was observed with increasing stress times (7DAS [0.50], 14DAS [0.48], and 21DAS [0.44]). Reduction in NDVI was significantly greater with 21 days of drought stress than with other stress times and in control plants (**Figure 1B**).

### Biochemical changes with drought stress

Biochemical profiling of leaves with drought stress treatments helps understand the stress responses and induced protective mechanisms in plants. We observed enhanced production of MDA in sugar maple with increasing drought stress time. The MDA content was significantly induced at 7DAS (1.14 µmoles/g FW) and showed an increasing trend with increasing stress time: 14DAS and 21DAS (1.39 and 1.71 µmoles/g FW, respectively) (**Figure 1C**). Among all the time points, control samples showed less MDA content (0.8 µmoles/g FW).

Therefore, we studied the accumulation of proline in sugar maple leaves in response to drought stress. Proline content showed an increasing trend with prolonged stress in sugar maple leaves. It was significantly higher at 21DAS (13.8 µmoles/g FW) than at other stress times (9.34 µmoles/g FW at 14DAS, and 7.35 µmoles/g FW at 7DAS) and control samples (4.79 µmoles/g FW) (**Figure 1D**).

### *De novo* transcriptome assembly and annotation

**Figure 2:**
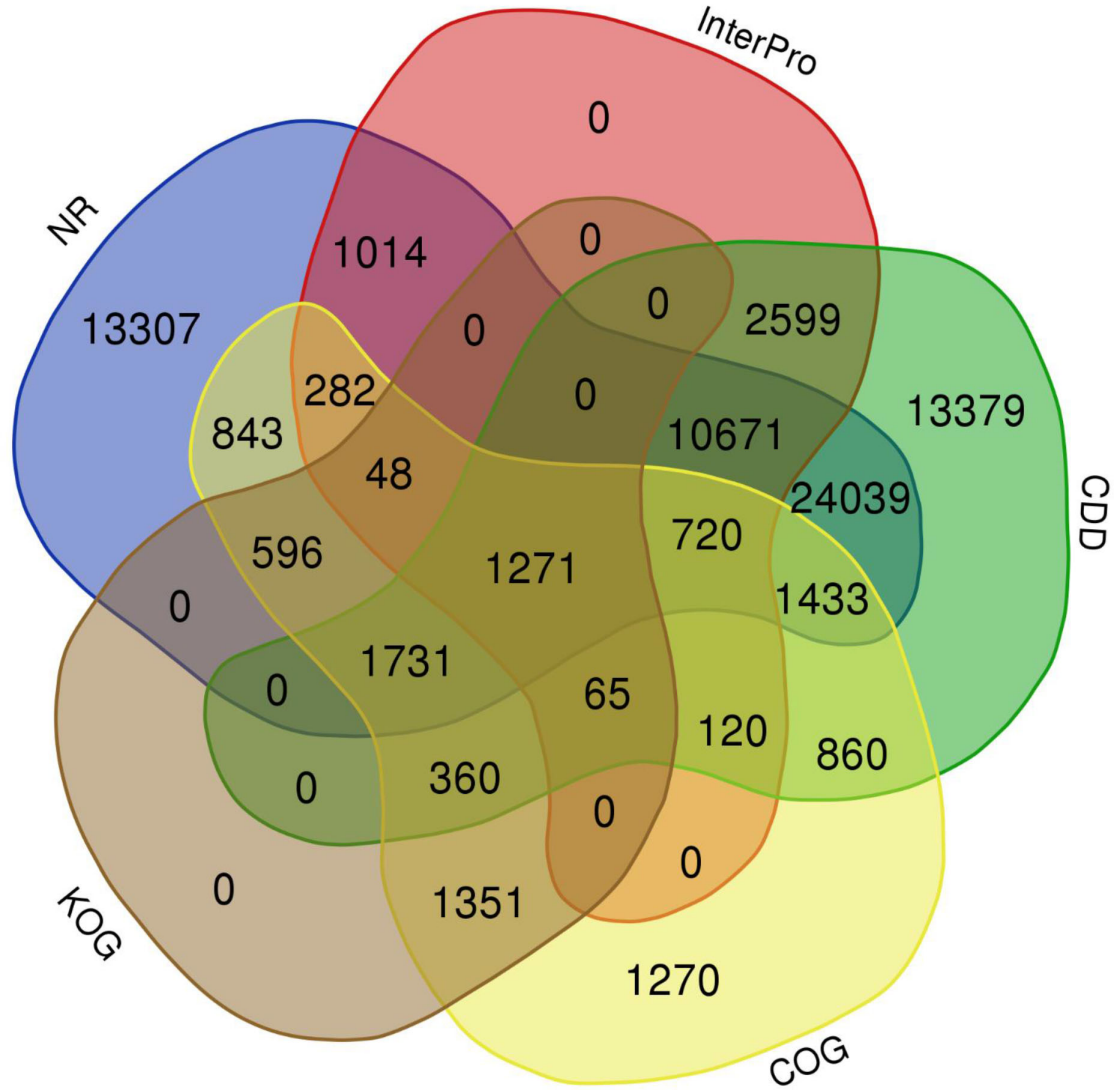
Venn diagram shows the annotation shared among NR, InterPro, CDD, COG, and KOG databases.

The quality filtered paired-end reads from the control and treatment samples (7, 14, and 21DAS) were assembled *de novo* using Trinity assembler. The *de novo* assembly and further clustering produced 310, 981 transcripts which included 218, 885 unique longest open reading frame (ORF) transcripts. The average contig size was 1,177, and the contig N50 was 2115 (**Table 1**). The resulting unigenes were used as a reference transcriptome for further analysis of RNAseq data. The completeness and integrity of the *de novo* transcriptome assembly from sugar maple were assessed using BUSCO analysis and the embryophyta_odb10 dataset. Of the 1614 BUSCOs from the embryophyta orthologue groups, 89% were identified as complete BUSCOs in the sugar maple transcriptome assembly, indicating the high-quality reference transcriptome data set comparable with transcriptome assembly from land plants (Gault et al., 2018). The assembly also contained 81.8% of the single-copy BUSCOs and only 5.8% of the missing BUSCOs (**Table 2**). A total of 55,955 unigenes were successfully annotated using the NR database at NCBI and including InterPro (UniProtKB/Swiss-Prot), COG, KOG, and CDD databases provided high confidence annotation to a total of 75,959 unigenes (**Table S2**; **Figure 2**).

**Table 1:** Summary of *de novo* transcriptome assembly.

	Number
Total number of transcripts	310,981
Total assembled bases	366,101,982
Percent GC	45.65
Average contig size	1,177
Contig N50	2,115

**Table 2:** Summary of BUSCO analysis.

Particulars	Number	Percentage
Complete BUSCOs (C)	1436	89%
Complete and single-copy BUSCOs (S)	1320	81.8%
Complete and duplicated BUSCOs (D)	116	7.2%
Fragmented BUSCOs (F)	84	5.2%
Missing BUSCOs (M)	94	5.8%
Total BUSCO groups searched	1614	

### Summary of RNAseq and mapping

RNAseq was performed under drought stress to assess the global transcriptome profile of sugar maple leaf samples. RNAseq of 16 samples with the four treatments and four biological replicates under “irrigated control” or “drought stress treatment (7, 14, and 21DAS)” generated a total of 395,240,587 raw reads; 365,445,423 were identified as filtered reads (**Table S3**). These samples were clustered based on the gene expression and presented with a principal component analysis (PCA) plot in **Figure S3**. The filtered reads were aligned and mapped to the reference *de novo* transcriptome with an average mapping percentage of 92%. The summary of RNAseq reads and genome mapping are given in **Table S3**.

### DEGs in response to drought stress

DEGs were determined between the control and the three different drought stress treatments in sugar maple leaf samples. The number of DEGs between the control and 7, 14, and 21DAS samples are shown in **Table S4** and were filtered using log2 fold-change cutoff ≥ 1 and FDR <0.05. The number of total DEGs was greater between 7DAS and the control (9,936); most were downregulated (9,520). The number of total DEGs was lowest among 14DAS and the control (2,374), and more were upregulated (1,223) than downregulated genes (1,151). The total number of DEGs between 21DAS and the control was 4,456: 2,978 were upregulated and 1,478 downregulated (**Table S4**). The overall differential gene expression pattern and their variation under specific drought stress regimes are visualized with a PCA plots and volcano plot in **Figures S3** and S4.

**Figure 3:**
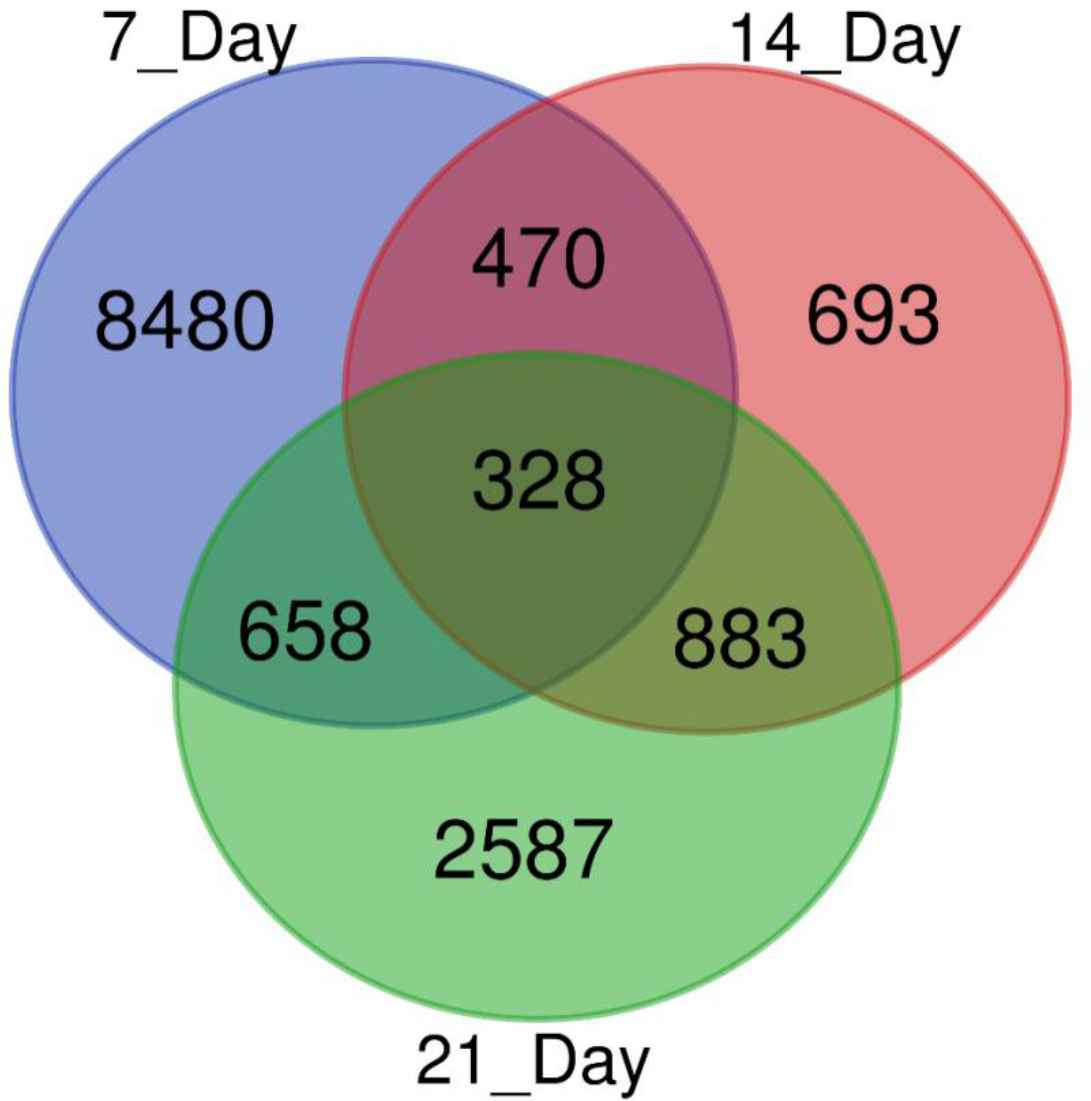
Venn diagram showing the number of DEGs obtained from different drought stress imposition periods in sugar maple.

We used a pair-wise comparison of the DEGs from three different drought stress conditions to explore unique as well as common DEGs in response to drought stress in sugar maple (**Figure 3**). A total of 14,099 DEGs were identified during drought stress against the control samples: 328 genes were common between all three drought stress times, and 8480, 639, and 2,587 were unique at 7, 14, and 21DAS, respectively. The gene number shared between 7DAS and 14DAS was 470, and between 7DAS and 21DAS was 658. The number shared between 14DAS, and 21DAS was 883. Lists of significantly up- or downregulated DEGs among all drought stress treatments and their annotations are in **Tables S5**-**S8**.

Among the 328 DEGs common to all stress times, a large number were related to the chloroplast, protein kinases, membrane transporters, and protein detoxification. Many genes encoded stress-responsive transcription factors, such as NAC, HSF, ZFPs, RING-finger, YTH, HD-Zip, GRFs, and ERF, which are involved in alleviating drought stress in plants (Joshi et al., 2016). In addition, DEGs included stress-responsive genes involved in antioxidant activity (peroxidases, dehydrogenases, reductases, cytochrome P450, PAL), sugar metabolism, splicing, ubiquitination, hormonal, and signaling (**Table S8**).

### GO assignments for functional classification of DEGs

**Figure 4:**
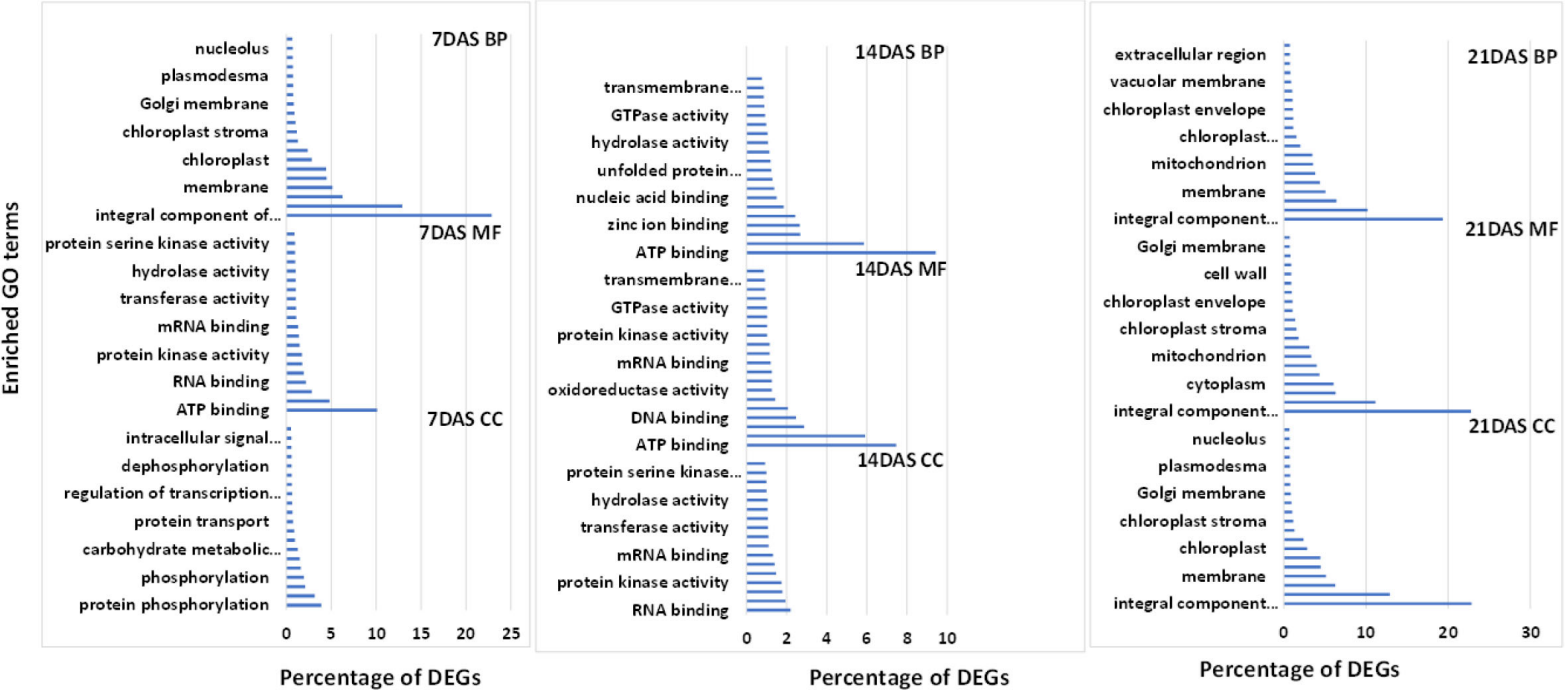
Top 20 enriched GO terms for the common DEGs in sugar maple leaf samples imposed to different drought stress periods (7, 14, and 21 days of drought stress). BP: biological process, CC: cellular component, and MF: molecular functions.

We used GO enrichment analysis of 14,099 genes to identify the enriched biological processes and functions in the DEGs. GO enrichment analysis is used for understanding the regulation of DEGs under the three essential categories: biological process (BP), molecular function (MF), and cellular component (CC). The top 20 enriched GO terms under each category (BP, MF, and CC) at different stress periods are in **Figure 4**. GO enrichment analysis of DEGs from the three different drought stress periods revealed the functional categories activated under drought stress treatments. Under BP, the highest percentage of DEGs commonly enriched with all three drought stress treatments included protein phosphorylation (2.5-3.9%) and transmembrane transport (2.4-3.1%). Among MF, the highest percentage of DEGs commonly enriched was ATP (7-10%), DNA (2-2.4%), RNA (2.2-2.8%), metal ion (4.8-5.9%), and zinc ion binding (1.9-2.6%). Among CC, the highest percentage of DEGs commonly enriched was an integral component of the membrane (19.3-22.8%), nucleus (10.1-12.9%), cytoplasm (6-6.4%), and membrane (5-6.3%) processes. The overall list of the GO enrichment process is in **Table S9**.

### KEGG pathway enrichment analysis of DEGs

A total of 249 KEGG terms were assigned to the 14,099 DEGs from all drought stress experiments. Overall, 56 KEGG pathways were significantly enriched (FDR-adjusted P < 0.05) with different drought stress periods (7, 14, and 21 days). Both up- and downregulated DEGs were involved in the metabolic pathways and biosynthesis of secondary metabolites pathway under all drought stress regimes (**Table 3**). The number of pathways in upregulated DEGs was highest at 21DAS and lowest at 7DAS. The number of pathways in downregulated DEGs was highest at 7DAS and lowest at 14DAS. The other most enriched pathways in both up- and downregulated DEGs included carbon metabolism, protein processing in the endoplasmic reticulum, circadian rhythm-plant, carotenoid biosynthesis, carbon fixation in photosynthetic organisms, fatty acid degradation glycerolipid metabolism, biosynthesis of amino acids, and flavonoid biosynthesis pathways (**Tables 3**, **S10**).

**Table 3:** KEGG pathway enrichment analysis of differentially expressed genes in response to drought stress in sugar maple.

S. No	#Term	ID	Drought Stress		
			7DAS	14DAS	21DAS
1	2-Oxocarboxylic acid metabolism	ath01210	.	↓	.	
2	Alanine, aspartate and glutamate metabolism	ath00250	.	↓	↓	
3	Arachidonic acid metabolism	ath00590	.	.	↑	
4	Arginine biosynthesis	ath00220	.	↓	↓	
5	Ascorbate and aldarate metabolism	ath00053	↓	.	↑	
6	beta-Alanine metabolism	ath00410	↓	.	.	
7	Biosynthesis of amino acids	ath01230	↓	↓	↓	
8	Biosynthesis of secondary metabolites	ath01110	↑↓	↑↓	↑↓	
9	Carbon fixation in photosynthetic organisms	ath00710	↓	↓	↓	
10	Carbon metabolism	ath01200	↓	↓	↓	
11	Carotenoid biosynthesis	ath04712	.	↑	.	
12	Circadian rhythm – plant	ath04712	↓	↑↓	↑↓	
13	DNA replication	ath03030	↓	.	.	
14	Endocytosis	ath04144	↑	↑↓	↑↓	
15	Fatty acid degradation	ath00071	↓	.	.	
16	Flavonoid biosynthesis	ath00941	↓	.	↓	
17	Folate biosynthesis	ath00790	.	↓	.	
18	Fructose and mannose metabolism	ath00051	.	.	↓	
19	Galactose metabolism	ath00052	.	.	↑↓	
20	Glycerolipid metabolism	ath00561	↓	↑	↑	
21	Glycine, serine, and threonine metabolism	ath00260	↓	↓	↓	
22	Glycolysis/Gluconeogenesis	ath00010	↓	↓	↓	
23	Glyoxylate and dicarboxylate metabolism	ath00630	↓	↓	↓	
24	Histidine metabolism	ath00340	↓	.	.	
25	Homologous recombination	ath03440	↓	.	.	
26	Inositol phosphate metabolism	ath00562	↓	.	.	
27	Lysine degradation	ath00310	↓	.	.	
28	Metabolic pathways	ath01100	↑↓	↑↓	↑↓	
29	Mismatch repair	ath03430	↓	.	.	
30	mRNA surveillance pathway	ath03015	↓	.	↑	
31	Nicotinate and nicotinamide metabolism	ath00760	↓	.	.	
32	Nitrogen metabolism	ath00910	.	↓	↓	
33	Nucleotide excision repair	ath03420	↓	.	.	
34	One carbon pool by folate	ath00670	↓	.	↓	
35	Other glycan degradation	ath00511	.	.	↑	
36	Pantothenate and CoA biosynthesis	ath00770	↓	.	.	
37	Pentose phosphate pathway	ath00030	↓	.	↓	
38	Peroxisome	ath04146	↓	.	.	
39	Photosynthesis	ath00195	.	.	↑	
40	Photosynthesis - antenna proteins	ath00196	.	.	↑	
41	Plant-pathogen interaction	ath04626	.	.	↑	
42	Porphyrin and chlorophyll metabolism	ath00860	↓	.	↑	
43	Propanoate metabolism	ath00640	↓	.	.	
44	Protein processing in endoplasmic reticulum	ath04141	↑	↑	↑	
45	Pyruvate metabolism	ath00620	↑↓	.	.	
46	Ribosome	ath03010	.	.	↑	
47	Ribosome biogenesis in eukaryotes	ath03008	↑	↑	↑	
48	RNA degradation	ath03018	↓	.	↑	
49	RNA polymerase	ath03020	.	.	↑	
50	RNA transport	ath03013	.	.	↑	
51	Spliceosome	ath03040	↓	.	↑↓	
52	Starch and sucrose metabolism	ath00500	.	.	↑↓	
53	Terpenoid backbone biosynthesis	ath00900	↑	.	.	
54	Ubiquinone and other terpenoid-quinone biosynthesis	ath00130	↓	.	.	
55	Ubiquitin mediated proteolysis	ath04120	↓	.	.	
56	Valine, leucine and isoleucine degradation	ath00280	↓	.	↑	

Upward arrow (↑) represents the significantly upregulated pathways and downward (↓) arrow represents the downregulated pathways. Both arrows (↑↓) represent the enrichment of both up- and downregulated pathways, and dots (.) indicate non-significant or not enriched pathways at a particular drought stress period. DAS: days after stress.

### Time series analysis of gene expression upon drought stress

**Figure 5:**
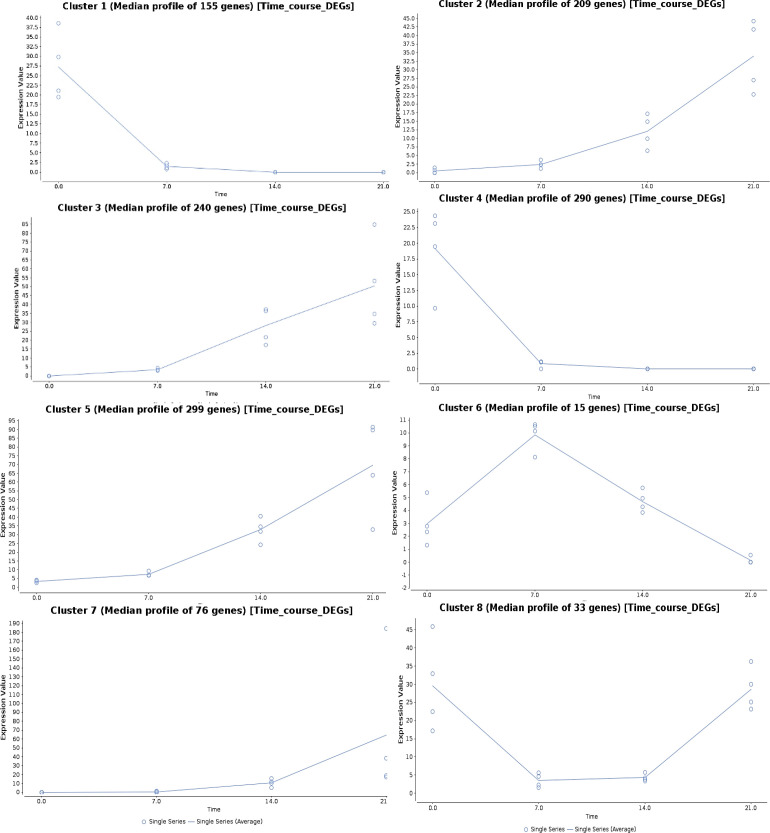
Time-series gene expression analysis showing the expression pattern in eight different clusters upon different drought stress periods in sugar maple.

The genes regulated under drought stress were clustered according to the expression ratios of drought stress samples to the control samples at each time point, which resulted in eight clusters based on their regulation pattern (**Figure 5**). The gene regulation in clusters 1 and 4 showed a downregulation pattern with increasing drought stress time, and the genes from four major clusters (2, 3, 5, and 7) showed an upregulation pattern. In cluster 6, the gene expression showed an increasing pattern at 7DAS, and with increasing stress periods at 14 and 21DAS, the expression showed a marked decrease. In cluster 8, the pattern of gene regulation was decreased at 7 to 14DAS and recovered with the increased regulation pattern at 21DAS. Eight major clusters, consisting of 1,317 genes, were classified into three groups: genes showing the upregulated pattern on drought stress (upregulated DEGs: clusters 2, 3, 5, and 7) and a downregulated pattern (downregulated DEGs: clusters 1 and 4) and a differential pattern (clusters 6 and 8), which contained 445,824 and 48 genes, respectively (**Figure 5**). We performed pathway enrichment analysis to identify significantly enriched stress pathway hubs individually from each of the five clusters. We identified 1, 4, and 9 significant KEGG pathways with FDR cutoff, P < 0.05 for clusters 2, 3, and 5 in the upregulated pattern and 12 and 18 pathways in clusters 1 and 4 in downregulated pattern, respectively. The significantly enriched pathways in clusters 2, 3, and 5 with the upregulated pattern are RNA degradation and transport, metabolic pathways, biosynthesis of secondary metabolites, Ascorbate and aldarate metabolism, porphyrin, and chlorophyll metabolism, circadian rhythm-plant, fatty acid degradation, and glycerolipid metabolism. The downregulated pattern that occurred in the cluster 1 and 4 showed the pathways such as carbon metabolism, carbon fixation in photosynthetic organisms, alanine, aspartate, and glutamate metabolism, metabolic pathways, biosynthesis of amino acids, biosynthesis of secondary metabolites, arginine biosynthesis, and glyoxylate and dicarboxylate metabolism (**Table S11**). Detailed cluster-wise information for all genes is in **Table S11**.

### Validation of DEGs expression by qRT-PCR and enzyme assay

**Figure 6:**
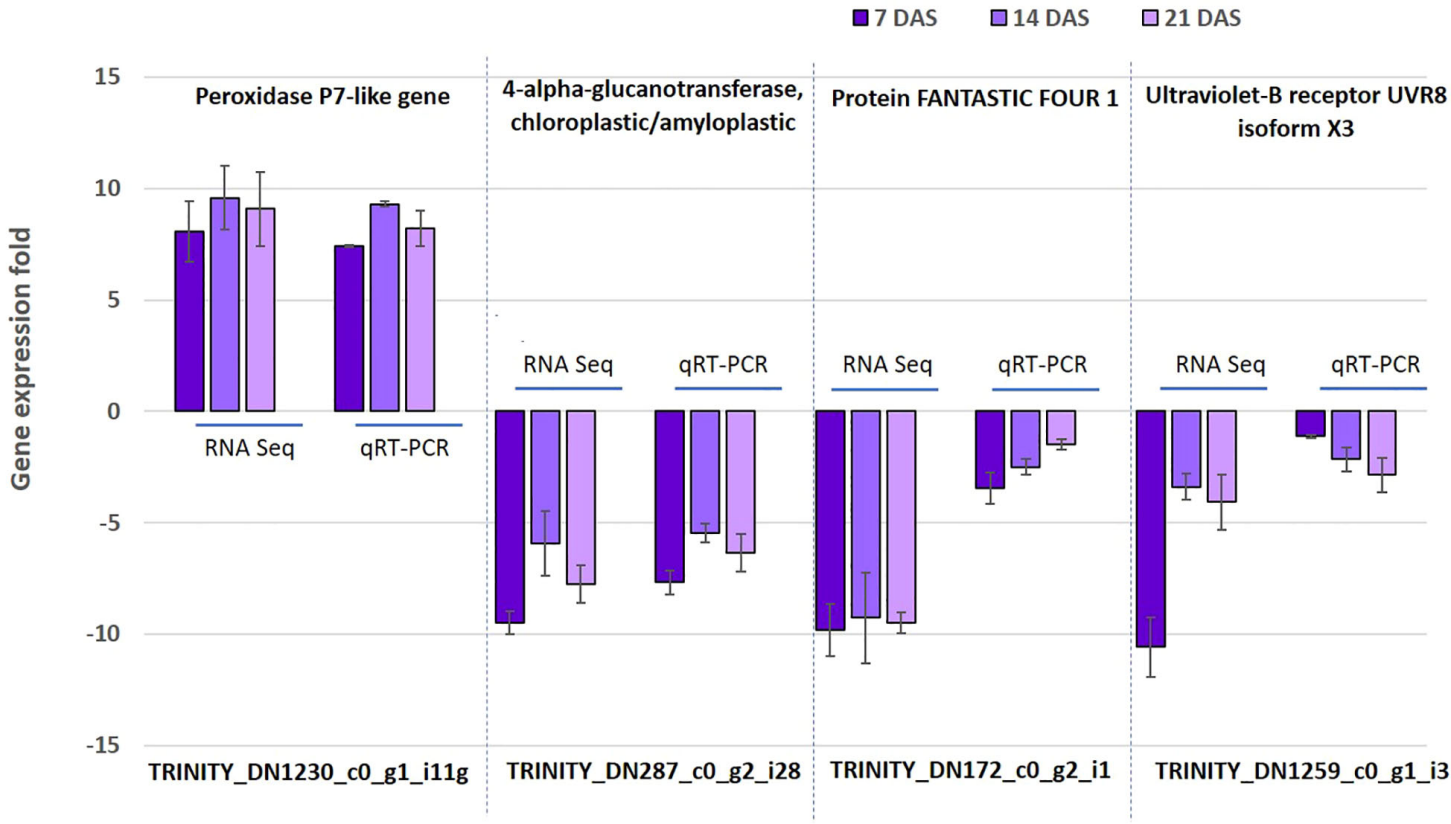
Validation of RNAseq data using quantitative real-time PCR. The expression of four selected genes was studied using qRT-PCR. Data are mean (SE) expressions from three biological replicates of sugar maple at each drought stress time. The *β-tubulin* and *actin* gene were used as an internal control for normalizing gene expression and fold change was calculated over the control.

To confirm the reliability and validity of the RNAseq results in sugar maple, we randomly selected four genes (TRINITY_DN1230_c0_g1_i11, TRINITY_DN287_c0_g2_i28, TRINITY_DN172_c0_g2_i1, and TRINITY_DN1259_c0_g1_i3) from DEGs for the 7, 14, and 21DAS samples for qRT-PCR analysis. The expression of the selected genes was positively correlated with the RNAseq results. Even though the detected fold change expression do not exactly match, the selected genes showed the same trends of expression patterns. For example, the upregulation of the peroxidase P7-like gene (TRINITY_DN1230_c0_g1_i11) and downregulation of the 4-alpha-glucanotransferase, chloroplastic/amyloplastic gene (TRINITY_DN287_c0_g2_i28) was confirmed by both RNAseq and qRT-PCR. Overall, the qRT-PCR expression followed a similar expression pattern of DEGs, thus confirming the consistency of the transcriptome sequencing analysis (**Figure 6**).

In order to assess the protein level expression of one of the top 10 upregulated DEGs (peroxidase P7-like gene), the peroxidase enzyme assay was performed. The increase in enzyme activity with increasing stress periods showed the activity of enzyme results is directly proportional to the gene expression data. However, no significant differences were observed in the activity of enzymes between the 14 and 21 DAS (**Figures 6**; **S5**).

## Discussion

Considering the increased demand for sugar maple and its multiple roles in providing ecosystem services across its range, understanding how sugar maple plants will be affected by climate change signifies the importance of handling our natural resources within the Anthropocene (Wolkovich and Cleland, 2014). This study describes the molecular mechanisms involved in the physiological and biochemical responses of sugar maple saplings to drought stress.

### Morpho-physiological signatures in response to drought stress

Perennial trees signal their water deficit with several symptoms. The most common alterations in appearance are lighter green to yellow-green foliage, scorches around the leaf margins, leaf wilting, necrosis, and drooping (Li et al., 2015; Ball, 2021; Ribeyre et al., 2022). We observed similar symptoms in sugar maple upon drought stress in the present study; severe symptoms occurred with increased drought stress time (**Figure S2**). The physiological indexes of reduced chlorophyll and NDVI observed in sugar maple under drought stress were consistent with the results of other drought stress studies of crop and tree species (Silber et al., 2003; Hassan and Ali, 2014; Li et al., 2015; Kargar et al., 2017; Basal and Szabo, 2020; Bissiwu et al., 2022) (**Figures 1A, B**).

### Biochemical responses of sugar maple to drought stress

MDA is an oxidative stress-induced product that acts as a biomolecular marker for cell membrane damage caused by lipid peroxidation injury in plants (Nisarga et al., 2017; Demirel et al., 2020). Its accumulation varies in response to drought stress depending on the length of stress exposure and stress intensity (Davey et al., 2005; Singh et al., 2017). Membrane lipid peroxidation also serves as an indicator of the prevalence of reactive oxygen species (ROS) and indicates the impact of stress on plant cell damage (Patel and Hemantaranjan, 2012; Nisarga et al., 2017; Zhang et al., 2021). We observed a marked increase in MDA level with increasing stress time in our sugar maple leaf samples (**Figure 1C**), which suggests that drought stress-induced oxidative stress caused lipid membrane damage in corroboration with prior reports.

The production of osmolytes like proline stabilizes membrane and protein structural damage under low leaf water potential conditions upon drought stress (Kiranmai et al., 2018). Osmolyte production varies according to stress severity, the length of stress exposure, and plant species (Anjum et al., 2011). Moreover, proline accumulation is a well-reported compatible osmolyte in plants in response to drought stress (Demirel et al., 2020). Our results showed higher levels of proline accumulation under drought stress conditions with prolonged stress in sugar maple (**Figure 1D**). Higher accumulation of proline in plants was involved in reducing photo-oxidative damage in chloroplast thylakoid membranes by scavenging and/or reducing ROS production (Verbruggen and Hermans, 2008; Kiranmai et al., 2018; Lokesh et al., 2019). Also, proline has been reported to stabilize and protect ROS scavenging enzymes and activate alternate detoxification pathways in plants upon exposure to various abiotic stresses (Ramu et al., 2016; Patykowski et al., 2018; Kijowska-Oberc et al., 2020; Nareshkumar et al., 2020). Therefore, induced proline content with increasing drought stress possibly acts as a direct antioxidant *via* the osmo-protecting mechanism as well as activating other antioxidant mechanisms.

### Molecular mechanisms in response to drought stress

Elucidating the gene-level stress adaptive features to overcome adverse environmental conditions is vital to understand the molecular mechanisms in response to drought stress in sugar maple. In this study, we identified 14,099 potential DEGs in response to drought stress; 328 were commonly expressed during all three drought-stress periods (**Figure 3**; **Table S5**-**S8**).

A number of DEGs common to all three stress periods that were related to chloroplast and photosynthesis were more frequently downregulated than upregulated in sugar maple, and similar patterns of DEGs were reported in other plant species in response to drought stress (Haider et al., 2017). Also, these results agree with reduced photosynthetic parameters (Chaves et al., 2009; Li et al., 2020). Many common DEGs encode protein kinases, which play crucial roles in stress sensing and signal transduction (Priya et al., 2019; Yang et al., 2019; Chen et al., 2021). Membrane transporter genes were differentially expressed in common DEGs; these genes play a key role in response to drought stress by transporting signaling molecules, ions, or osmolytes (Jarzyniak and Jasiński, 2014). We found the downregulation of protein detoxification genes (multidrug and toxic compound extrusion [MATE] transporters). These enzymes regulate plant growth and development processes by transporting secondary metabolites, toxic cell compounds, and hormone regulation (Du et al., 2021; Wang et al., 2022).

Transcription factors play an important role in signal transduction pathways in plants during stress by regulating the expression of downstream target genes *via* specific binding to cis-acting elements. NAC, bZIP, MYB, HSF, GRFs and ERF, WRKY, DREB, and ERFs are well-known transcription factor families that are responsive to drought stress (Kiranmai et al., 2018; Lokesh et al., 2019; Yang et al., 2019). In this study, transcription factor genes including NAC, HSF, ZFPs, YTH, HD-Zip, GRFs, and ERF were commonly expressed under drought stress (**Table S5**-**S8**). Among the transcription factor genes identified as being differentially expressed in the present study, many are known to play a key role in drought tolerance (Alves et al., 2011; Omidbakhshfard et al., 2015; Zhu et al., 2019; Sun et al., 2020; Yao et al., 2021). Furthermore, a plethora of drought stress tolerance genes differentially expressed in sugar maple included peroxidases, ubiquitin, cytochrome P450, hormone, lipid, antioxidant, and sugar metabolism-related and other stress-responsive genes (**Table S8**).

The unavailability of water induces a wide range of physiological and biochemical changes in plants. These responses include regulation of the cellular process, metabolic functions, and maintenance of photosynthesis, respiration, and oxidative damage of the cellular components under stress conditions in plants (Shinozaki & Yamaguchi-Shinozaki, 2007; Gao et al., 2018). Similar responses were observed in the present study. We identified a number of DEGs involved in pathways such as protein phosphorylation, photosynthesis, chloroplast organization, and their integral parts of stroma and thylakoid membrane. This observation shows the effect of drought stress on chlorophyll synthesis or photosynthetic activities and transcript level responses as evidenced by the reduced physiological indices like chlorophyll index and NDVI with increasing drought stress. The higher percentage of DEGs from transmembrane transport, ATP binding, metal and zinc ion binding, and integral component of membrane pathways suggest the drought-induced effect on membrane damage and gene-level response to overcome the effects of stress. This finding is also evidenced by the increased lipid peroxidation of the membrane in sugar maple upon drought stress (**Figure 1C** and **Table S8**).

A high number of DEGs involved in protein phosphorylation, carbohydrate metabolic process, oxidoreductase activity, and cytoplasm suggests the cellular level response of the sugar maple to overcome the oxidative stress induced by increasing drought stress, which is also corroborated by the enhanced production of osmolytes (**Figure 1D**). GO terms enriched in processes such as ATP, DNA, and RNA binding, nucleus, DNA-binding transcription factor activity, nucleotide binding, nucleic acid binding, and mRNA binding suggests the stress-induced gene signaling and molecular level response in sugar maple to drought stress (**Figure 4** and **Table S9**). Similar GO terms in response to drought stress were reported in other plant species as well as the expression of a high percentage of DEGs involved in photosynthesis, respiration, and oxidative damage (Estravis-Barcala et al., 2020; Sobreiro et al., 2021; Tahmasebi and Niazi, 2021).

Metabolic pathways and biosynthesis of secondary metabolites were the most commonly expressed pathways at 7, 14, and 21 days of drought stress and were significantly enriched both in up- and down-regulated DEGs. Other pathways such as carbon metabolism, circadian rhythm-plant, carotenoid biosynthesis, glycerolipid metabolism, and protein processing in the endoplasmic reticulum were significantly enriched, which suggests that these pathways were activated under drought stress. Induced and downregulated DEGs involved in metabolic pathways, carotenoid biosynthesis, circadian rhythm, carbon metabolism, porphyrin and chlorophyll metabolism, photosynthesis-antenna proteins, photosynthesis, and carbon fixation in photosynthetic organism pathways suggesting that the photosynthetic response copes with stress-induced damage on chlorophylls and other pigments, as evidenced by the reduced SPAD metric and NDVI in sugar maple (**Table 3**, **Table S10**). Significantly enriched DEGs in metabolic pathways, glycerolipid metabolism, fatty acid degradation, glycolysis/gluconeogenesis, and protein processing in the endoplasmic reticulum were upregulated possibly to protect the lipid membrane protein damage caused by the induced lipid peroxidation upon drought stress. Significantly enriched DEGs in metabolic pathways, biosynthesis of amino acids, biosynthesis of secondary metabolites, pentose phosphate pathway, starch and sucrose metabolism, fructose, and mannose metabolism pathways may induce osmolyte production for protecting cellular level damage in sugar maple in response to drought stress. DEGs enriched in flavonoid biosynthesis, folate biosynthesis, beta-alanine metabolism, and biosynthesis of secondary metabolite pathways suggest the activation of antioxidant machinery under drought stress in sugar maple.

In addition to nonenzymatic antioxidative machinery plants have developed various enzymatic antioxidative detoxification mechanisms in adoption to drought stress. Antioxidant enzymes such as peroxidase, ascorbate peroxidase, superoxide dismutase, and catalase play major roles in ROS scavenging molecules and minimize their levels under drought stress (Sharma and Zheng, 2019; Wei et al., 2019; Li et al., 2023). In our study, many DEGs related to antioxidative activity, peroxidases, and other detoxification enzymes like cytochrome P450 suggest the important role of enzymes in ROS scavenging in response to drought stress (**Table S8**). Significantly enhanced expression of peroxidase at both gene and protein (enzyme activity) levels suggests the substantial role of sugar maple in coping with drought stress (**Table S8** and **Figure S5**). We found a gene (TRINITY_DN1230_c0_g1_i11g) that encodes peroxidase-like protein was significantly upregulated at all the drought stress periods (7, 14, and 21 DAS) and maintained enhanced enzyme activity suggesting that ROS scavenging system plays a key role in protecting the sugar maple from drought stress.

Time-series transcriptomic analysis allows for investigating the dynamic gene responses to drought stress and characterizing drought-responsive genes with respect to increasing drought stress and relevance to drought tolerance. Time-series gene expression analysis revealed different functional groups of genes regulated at 7, 14, and 21 days of drought (**Table S11**). Time series analysis was used in previous studies to understand the gene regulation patterns in response to drought stress in plants (He et al., 2020; Yi et al., 2022). Some plant physiological functions were suppressed, and a series of responses were activated to overcome the drought stress effects by complex gene regulatory and networks, as evidenced by global transcriptomic changes in eight clusters of regulation patterns we found in sugar maple (**Table S11**). Our results indicate that drought stress remarkably altered the expression of chlorophyll/photosynthesis-related and plant cell membrane and osmotic adjustment related genes to avoid cell damage in sugar maple. Overall, the results suggest that drought stress-induced changes in photosynthesis and cell membrane damage are possibly encountered by both enzymatic (peroxidase) and nonenzymatic antioxidant (proline) mechanisms in sugar maple saplings.

## Conclusion

The present study reports for the first time the characterization of the global transcriptome of sugar maple in response to drought stress. A set of physiological and biochemical traits examined in this study showed distinct signatures upon drought stress, suggesting that changes in chlorophyll biosynthesis, lipid membrane damage, and osmolyte production play a pivotal role in determining the sugar maple response to the severity of drought stress. The 328 common DEGs identified under all three drought stress periods in sugar maple will be a potential source for understanding the drought-tolerant mechanisms. Genes related to chlorophyll and carotenoid biosynthesis, membrane, oxidative stress response, osmolytes, and secondary metabolite production related to antioxidant activity pathways were significantly enriched on GO and KEGG analyses. These genes may play important roles in sugar maple adaptation or tolerance to the oxidative stresses caused by drought. In addition, we demonstrated the time-series expression profiling of global genes in response to drought stress for three time periods and identified the stress-responsive pathways. Overall, the study results help in understanding the drought stress-responsive molecular mechanisms complemented by physiological and biochemical responses of sugar maple and expand this knowledge in other tree species.

## Data availability statement

The original contributions presented in the study are publicly available. This data can be found here: https://www.ncbi.nlm.nih.gov/bioproject/895722.

## Author contributions

KM and SE conceived and designed the experiments. LM conducted the major experiments. AV, OJ and KK performed qRT-PCR. AV conducted a statistical analysis and summarized the transcriptome data. AV and LM wrote the manuscript. UR and PN performed RNAseq analysis. KM, KK, and SE revised the manuscript. All authors contributed to the article and approved the submitted version.

## Footnotes

1
https://agriculture.vermont.gov/sites/agriculture/files/documents/AgDevReports/Maple%20Syrup%20Market%20Research%20Report.pdf.

## Supplementary material

Supplementary Table 3 Summary of RNAseq and mapping analysis.

Supplementary Table 9 GO enrichment analysis.
