# Supplementary material for: Transcriptome profiling, physiological, and biochemical analyses provide new insights towards drought stress response in sugar maple (Acer saccharum Marshall) saplings
Source: Front Plant Sci. 2023 Apr 19;14:1150204. doi: 10.3389/fpls.2023.1150204 (PMC10154611; doi:10.3389/fpls.2023.1150204)
Supplement: Supplementary file 1 [file DataSheet_1.docx]

**Figure S1:** Soil moisture content measured with a TDR probe (a hand-held Field Scout TDR 100 System; Spectrum Technologies, Wales, UK) under different drought stress treatments (0, Control, 7, 14, and 21 days of drought stress).
