# Supplementary material for: Transcriptome profiling, physiological, and biochemical analyses provide new insights towards drought stress response in sugar maple (Acer saccharum Marshall) saplings
Source: Front Plant Sci. 2023 Apr 19;14:1150204. doi: 10.3389/fpls.2023.1150204 (PMC10154611; doi:10.3389/fpls.2023.1150204)
Supplement: Supplementary file 2 [file DataSheet_2.docx]

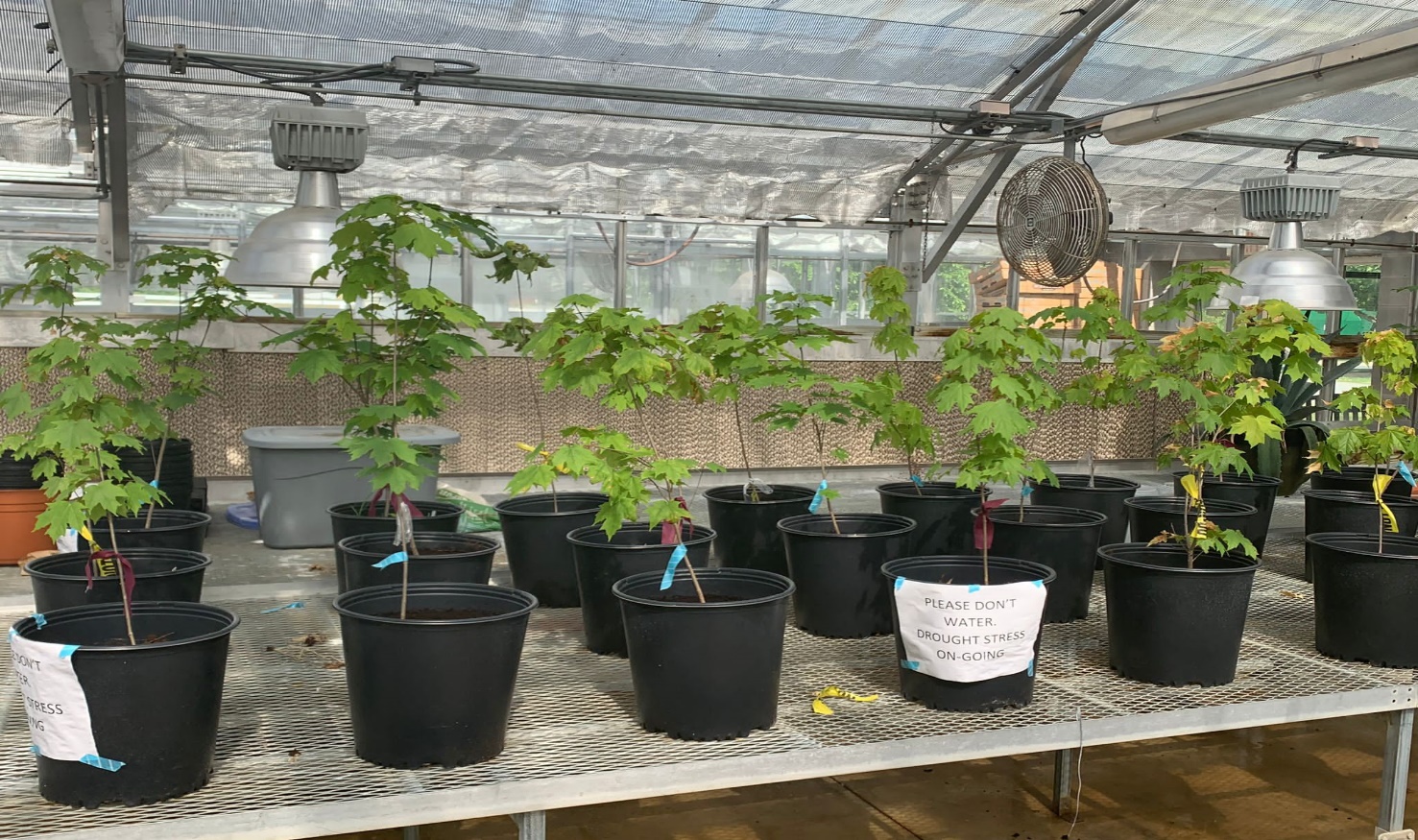


**A)**


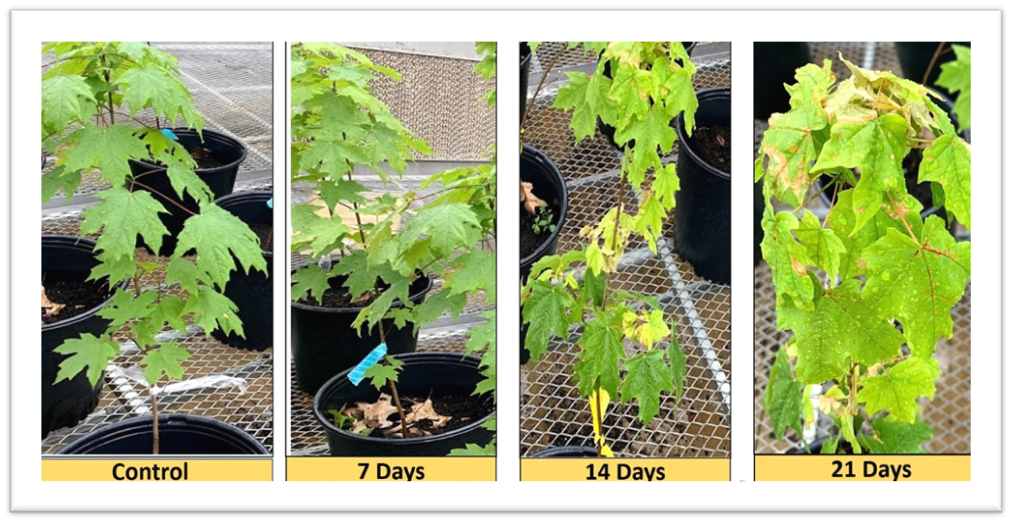


**B)**

**Figure S2:** Morphological changes in sugar maple during different times of drought stress. A). Before drought stress & B) after the drought stress imposition.
