# Supplementary material for: Transcriptome profiling, physiological, and biochemical analyses provide new insights towards drought stress response in sugar maple (Acer saccharum Marshall) saplings
Source: Front Plant Sci. 2023 Apr 19;14:1150204. doi: 10.3389/fpls.2023.1150204 (PMC10154611; doi:10.3389/fpls.2023.1150204)
Supplement: Supplementary file 3 [file DataSheet_3.docx]

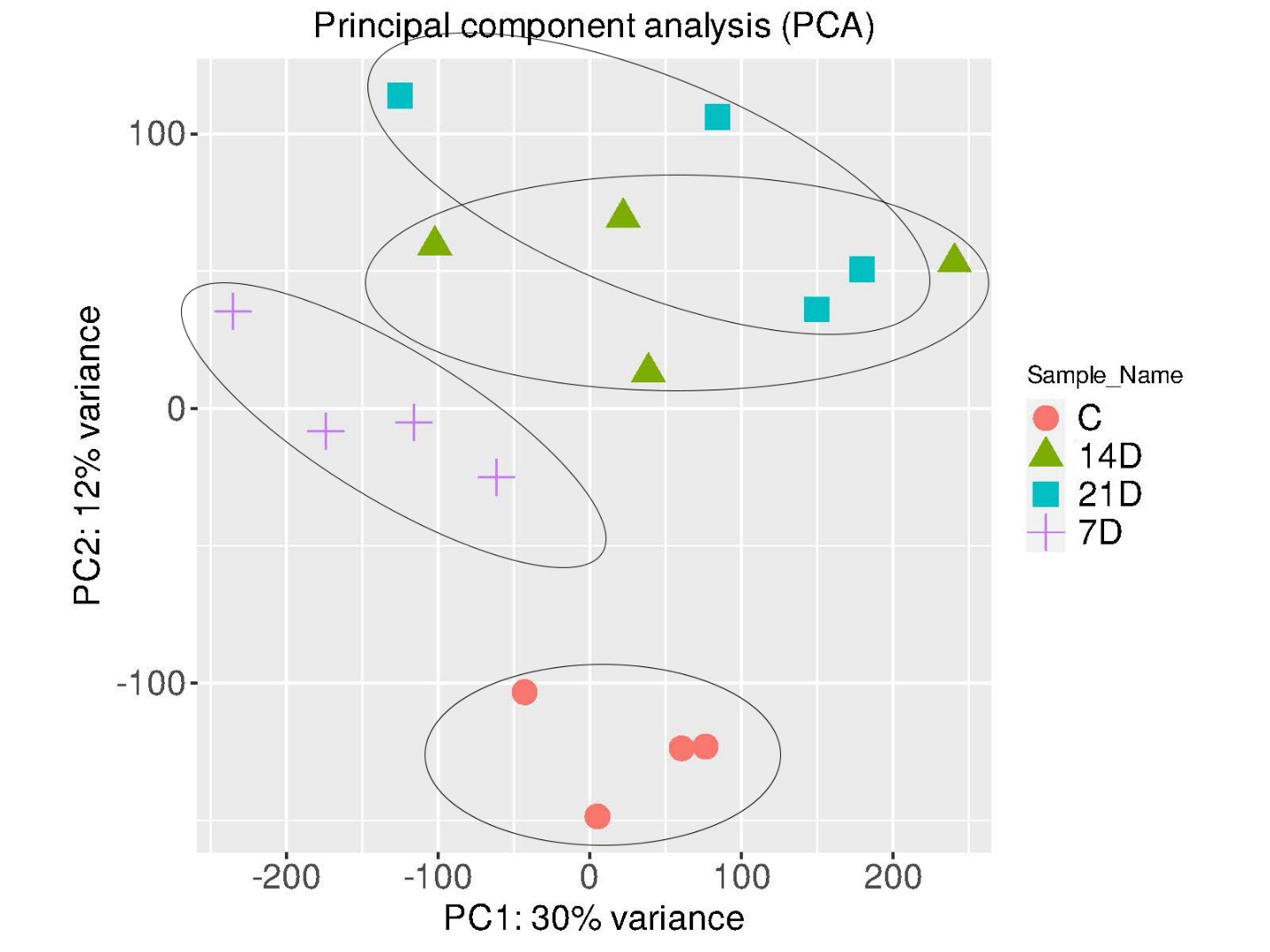


**Figure S3.** Principal Component Analysis (PCA) plots of samples from different treatment conditions showing clustering patterns. Samples with similar expressions are grouped together, and the groups are presented with different colors and shapes.
