# Supplementary material for: Transcriptome profiling, physiological, and biochemical analyses provide new insights towards drought stress response in sugar maple (Acer saccharum Marshall) saplings
Source: Front Plant Sci. 2023 Apr 19;14:1150204. doi: 10.3389/fpls.2023.1150204 (PMC10154611; doi:10.3389/fpls.2023.1150204)
Supplement: Supplementary file 4 [file DataSheet_4.docx]

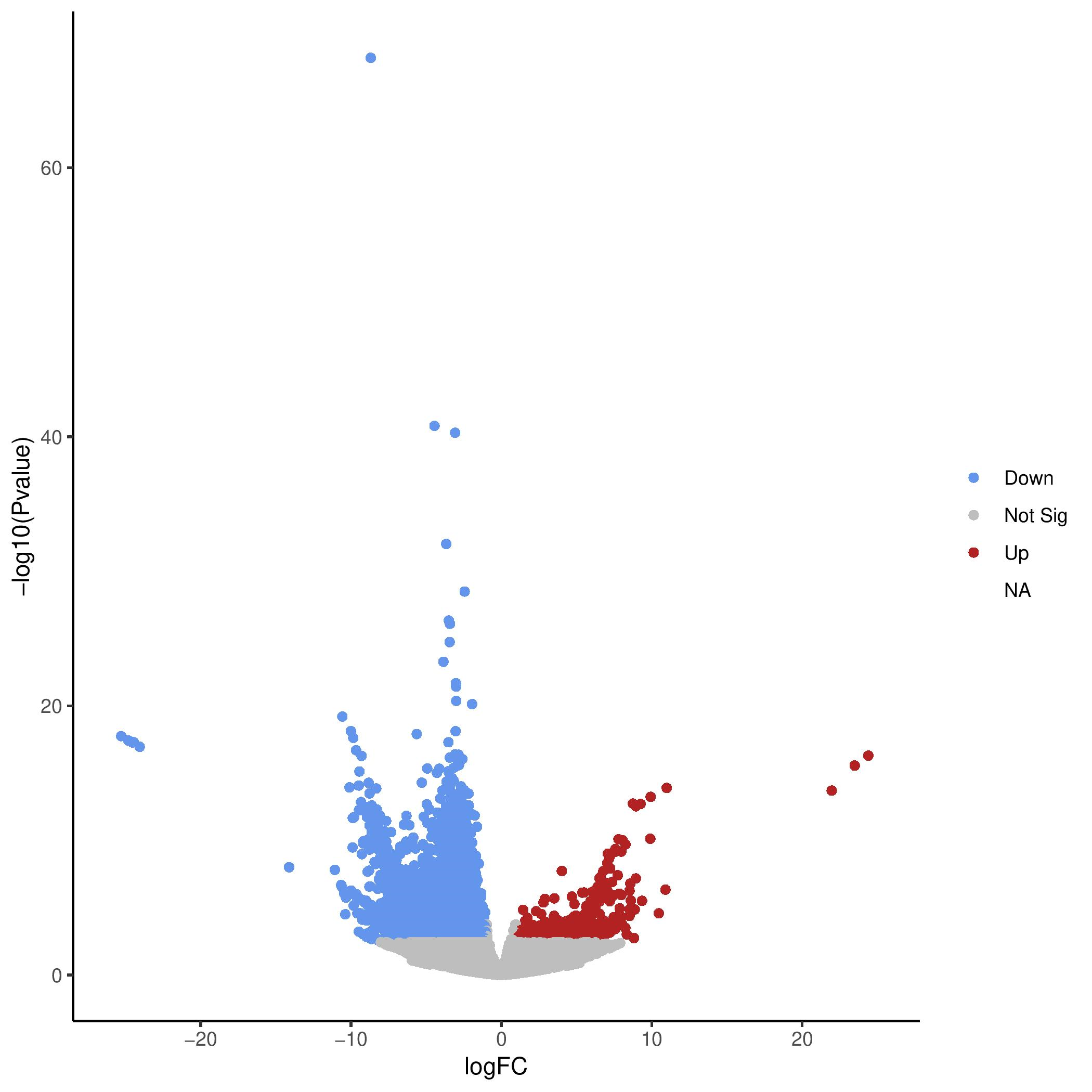

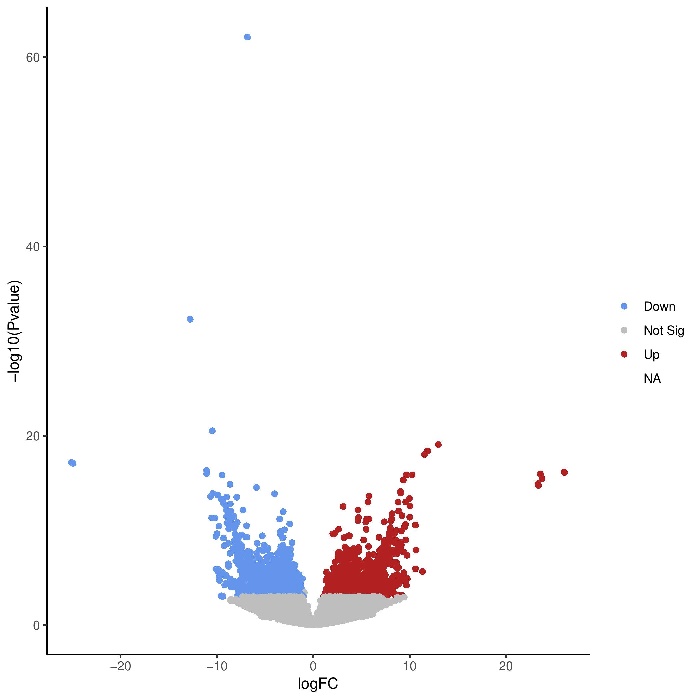

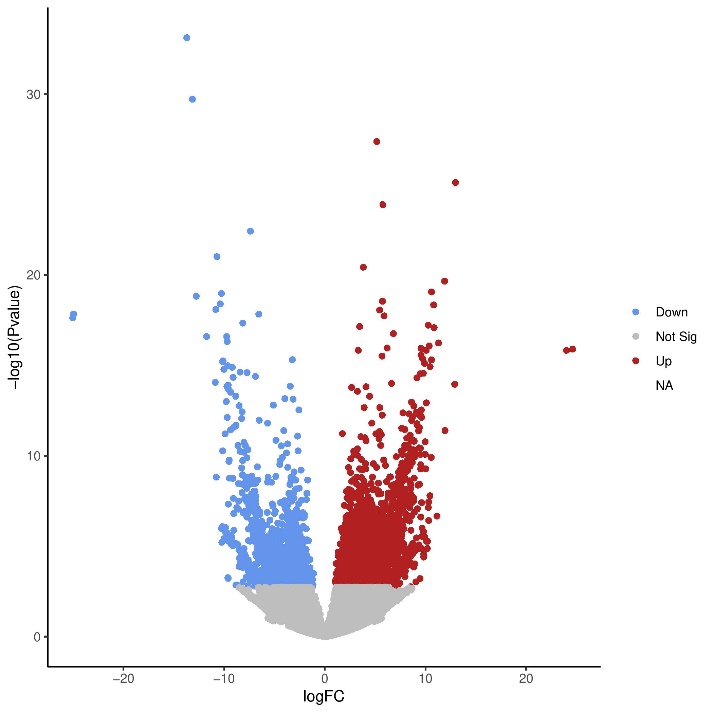


(A)

(B)

(C)


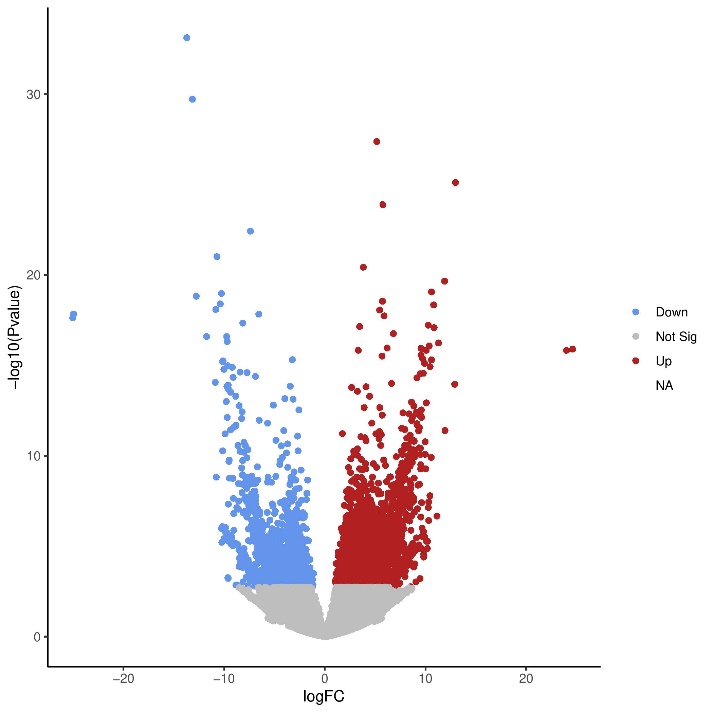


**Figure S4:** Volcano plot showing differentially expressed genes at (a) 7, (b) 14, and (c) 21 days of drought stress.
