# Supplementary material for: Transcriptome profiling, physiological, and biochemical analyses provide new insights towards drought stress response in sugar maple (Acer saccharum Marshall) saplings
Source: Front Plant Sci. 2023 Apr 19;14:1150204. doi: 10.3389/fpls.2023.1150204 (PMC10154611; doi:10.3389/fpls.2023.1150204)
Supplement: Supplementary file 5 [file DataSheet_5.docx]

**Figure S5:** Guaiacol peroxidase (POD) activity in sugar maple saplings subjected to different drought stress periods (7, 1,4, and 21 days after drought stress).
